# High prevalence of *Histoplasma capsulatum* in bats and pigeons is linked to human histoplasmosis in an endemic area of Ecuador

**DOI:** 10.3389/fvets.2025.1613841

**Published:** 2025-09-09

**Authors:** Naomi Mora-Jaramillo, Solón Alberto Orlando, Mireya Rivera, José Echevarría, Elsy Carvajal, Sebastián Rodríguez-Pazmiño, Darwin Santiago Paredes, Fabricio Arcos Alcivar, Valeria Rebolledo, Tais Fuentes, Odalys Delgado, Pamela Valencia, Mirna Oviedo, Evelyn Barona Moran, Henry Parra Vera, Miguel Angel Garcia-Bereguiain

**Affiliations:** ^1^Instituto Nacional de Salud Pública e Investigación, Guayaquil, Ecuador; ^2^Universidad Espíritu Santo, Guayaquil, Ecuador; ^3^Universidad Católica Santiago de Guayaquil, Guayaquil, Ecuador; ^4^One Health Research Group, Universidad de Las Américas, Quito, Ecuador; ^5^Universidad Ecotec, Guayaquil, Ecuador; ^6^Universidad Agraria del Ecuador, Guayaquil, Ecuador; ^7^Universidad Técnica de Manabí, Portoviejo, Ecuador; ^8^Ministerio de Ambiente y Agua, Guayaquil, Ecuador; ^9^Centro de Investigación Microbiológica, Guayaquil, Ecuador

**Keywords:** histoplasmosis, *Histoplasma capsulatum*, pigeons, bats, One Health, Ecuador

## Abstract

Histoplasmosis, a systemic mycosis caused by the fungal pathogen *Histoplasma capsulatum,* is a global public health concern, particularly in immunocompromised patients. The global burden of this disease is estimated in 500,000 people affected annually with around 100,000 cases progressing to disseminated histoplasmosis, the most severe clinical form of the disease with 30–50% mortality rate in treated patients. Histoplasmosis is very endemic in areas associated with river valleys. In South America, histoplasmosis is one of the most important causes of mortality in HIV patients, accounting for approximately 30% of deaths in this population, and it is frequently misdiagnosed. The animal reservoirs for these pathogens include bats and birds like pigeons, and fecal deposition in areas with high density of those animals represents hotspots of fungal contamination. In this work, we studied the prevalence of *H. capsulatum* in bats and pigeons from Guayas Province in Ecuador by PCR. A total number of 61 pigeons and 213 bats samples were collected, and the overall prevalence was 13.11% (95%CI: 10.54–15.68) and 21.14% (95%CI: 17.00–25.28), respectively. Moreover, Sanger sequencing analysis was carried out for *hcp100* gene, followed by phylogenetic analysis including 17 sequences isolated from human patients in Guayaquil city (Guayas Province). Our results show that bats and pigeons are reservoirs for *H. capsulatum* linked to transmission to humans in Guayas Province. An integrative One Health surveillance and control program including animal reservoirs should be improved to reduce the burden of histoplasmosis, especially considering the high burden of HIV in Guayaquil city.

## Introduction

Histoplasmosis, a systemic mycosis caused by the dimorphic fungal pathogen *Histoplasma capsulatum,* is a global public health concern, particularly in immunocompromised patients (1, 2). It is estimated that close to 500,000 people are affected by this disease annually in the world, with around 100,000 cases progressing to disseminated histoplasmosis, the most severe clinical form of the disease (1, 3). In such cases, mortality rate ranges from 30 to 50% in treated cases and about 100% in untreated cases (1). Histoplasmosis is very endemic in areas associated with river valleys, particularly in the Central and Eastern United States, where it is estimated that 60–90% of population has been exposed to this pathogen (4). Moreover, in Central and South America, especially in countries like Brazil, Venezuela, Ecuador, Paraguay, Uruguay, and Argentina, histoplasmosis is one of the most important causes of mortality in HIV patients, accounting for approximately 30% of deaths, and is frequently misdiagnosed due to its nonspecific clinical presentation, which often overlaps with tuberculosis (4–7).

*H. capsulatum* includes multiple genetically distinct clades with geographic and ecological specificity (8–11). Based on whole genome sequencing (WGS) or multi locus sequence typing (MLST), at least eight phylogenetic groups are recognized, including North American (NAm 1 and NAm 2), Latin American (LAm A and LAm B), Australian, Eurasian, African (*H. capsulatum* var. duboisii), and Dutch clades (8, 10, 12, 13). Moreover, recent studies have revealed regionally relevant sub clades in Latin America, like the “RJ sub clade” described in Brazil, highlighting the genus’s remarkable genetic variability (9, 10, 14).

In endemic areas, *H. capsulatum* thrives in ecological niches rich in organic matter, particularly in environments contaminated with guano from bats (especially cave-dwelling species) or droppings from pigeons, starlings and chickens (4, 15, 16). For instance, high-density bat colonies in caves amplify guano deposition, leading to localized hotspots of fungal contamination (17). Birds and bats are asymptomatic carriers and their droppings contribute to soil nitrogen enrichment, further promoting fungal proliferation (18, 19). Migratory bats and birds also play a significant role in the geographic spread of *H. capsulatum*, transporting the fungus across regions during seasonal movements (9, 19, 20).

In Ecuador, histoplasmosis is the most frequent fungal disease in HIV patients with an estimated burden of 1,110 cases nationwide (21). Guayas Province is an endemic zone due to its tropical climate, urban–rural interface and environmental exposure risk (22). Moreover, this province has the highest HIV prevalence in Ecuador, a risk population for histoplasmosis with estimations of 11.1% affected HIV patients (21). Urbanization and land-use changes, including agricultural activities and urban construction, disrupt fungal reservoirs, creating overlapping exposure zones that heighten the risk of infection for both rural and urban populations (20, 22). Despite the significant burden of histoplasmosis in endemic regions like Ecuador, this disease remains neglected with limited surveillance and diagnostic capabilities (1, 21). Misdiagnosis, particularly due to overlapping clinical symptoms with other diseases like tuberculosis, and the lack of specific diagnostic tools such as molecular testing, contribute to underreporting and delayed treatment (23–25). Moreover, there is no information about animal reservoirs and transmission dynamics of *H. capsulatum* in Ecuador to improve surveillance and control strategies to target risk populations based on a One Health approach.

The aim of this work was to study the prevalence of *H. capsulatum* in bats from Guayas Province and pigeons from the city of Guayaquil in Ecuador, and to carry out an integrative phylogenetic analysis including human cases from this area to better understand animal-human transmission.

## Methodology

### Study area and sample collection

**Figure 1:**
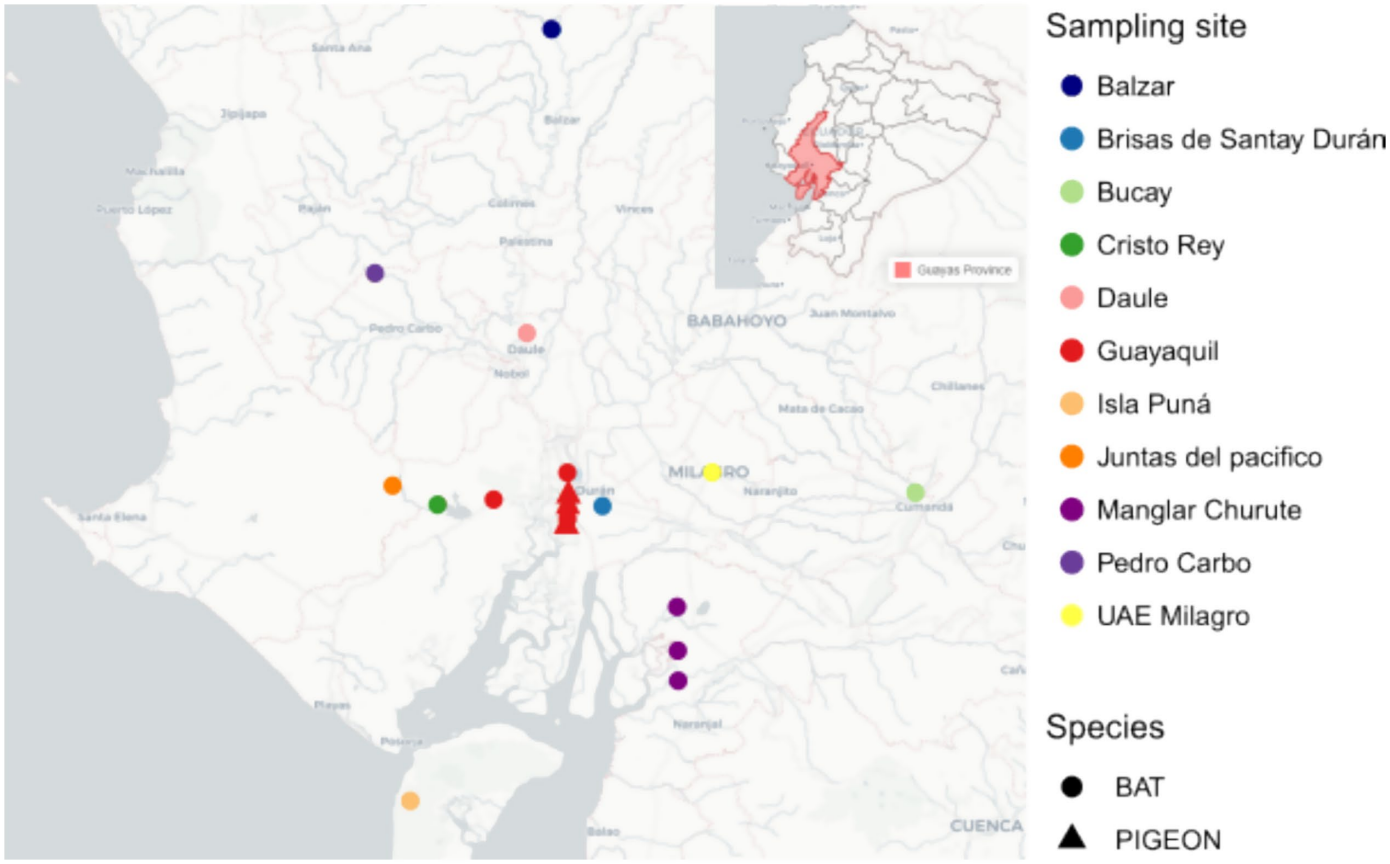
Geographic distribution of bat and pigeon sampling sites in Guayas Province, Ecuador.

A total number of 213 bats belonging to 21 different species were collected from October 2022 to May 2024, from 11 diverse geographical locations within Guayas Province in the Coastal Region of Ecuador (Figure 1). The species of bat included in the study were *Molossus molossus, Carollia perspicillata, Glossophaga soricina, Carollia castanea, Sturnira bakeri, Artibeus lituratus, Anoura peruana, Carollia brevicauda, Desmodus rotundus, Artibeus fraterculus, Artibeus concolor, Artibeus rosenbergi, Chiroderma trinitatum, Eptesicus innoxius, Micronycteris megalotis, Myotis nigricans, Phylloderma stenops, Phyllostomus discolor, Phyllostomus hastatus, Rhogeesa velilla,* and *Saccopteryx bilineata.* For details about the distribution of bats across species see Table 1. A convenience sampling approach was employed to cover several urban locations in Guayaquil city and rural communities within Guayas Province. The sampling campaign included night fieldwork and the bats were captured using mist nets and euthanized by cervical dislocation. For risk factor analysis, several variables were considered for each bat captured: (1) animal sex (male or female); (2) origin of the animal, considering two categories: (a) bats collected in urban settings within Guayaquil city; (b) bats collected in rural communities within Guayas Province; (3) capture zone, referring to nine different categories where bats were hunted: forest, park, house, cave, river, livestock pen, tree, warehouse, and orchard.

**Table 1:** Prevalence of *Histoplasma capsulatum* in bats and pigeons detected by PCR.

Species	Urban (*n*)	Rural (*n*)	Total sample size (*n*)	Prevalence per species (%)	95% CI
*Molossus molossus*	*60*	*48*	108	17.59	(14.14–21.04)
*Carollia perspicillata*	*0*	*26*	26	26.92	(21.65–32.20)
*Glossophaga soricina*	*0*	*14*	14	35.71	(28.71–42.71)
*Carollia castanea*	*0*	*7*	7	42.86	(34.46–51.26)
*Sturnira bakeri*	*0*	*7*	7	42.86	(34.46–51.26)
*Artibeus lituratus*	*0*	*3*	3	66.67	(53.60–79.73)
*Anoura peruana*	*0*	*4*	4	50.00	(40.20–59.80)
*Carollia brevicauda*	*0*	*9*	9	22.22	(17.87–26.58)
*Desmodus rotundus*	*0*	*4*	4	25.00	(20.10–29.90)
*Artibeus fraterculus*	*0*	*11*	11	9.09	(7.31–10.87)
*Artibeus concolor*	*0*	*1*	1	0.00	-
*Artibeus rosenbergi*	*0*	*1*	1	0.00	-
*Chiroderma trinitatum*	*0*	*3*	3	0.00	-
*Eptesicus innoxius*	*0*	*2*	2	0.00	-
*Micronycteris megalotis*	*0*	*1*	1	0.00	-
*Myotis nigricans*	*0*	*1*	1	0.00	-
*Phylloderma stenops*	*0*	*2*	2	0.00	-
*Phyllostomus discolor*	*0*	*1*	1	0.00	-
*Phyllostomus hastatus*	*0*	*1*	1	0.00	-
*Rhogeesa velilla*	*0*	*2*	2	0.00	-
*Saccopteryx bilineata*	*0*	*2*	2	0.00	-
Not determined	0	3	3	0.00	-
Total Bats	**60**	**153**	**213**	**21.14**	**(15.43–26.85)**
Pigeons	**61**	**0**	**61**	**13.11**	**(10.54–15-68)**

A total number of 61 pigeons (*Columba livia*) were collected in the city of Guayaquil (Ecuador), between August 2023 and April 2024. Sampling was conducted at convenience in various urban zones, including areas where pigeons were found nesting in infrastructure or parks. Pigeons were captured using mist nets and euthanized by cervical dislocation. For risk factor analysis, two variables were considered for each pigeon captured: (1) animal sex (male or female); (2) capture zone, considering two options: (a) pigeons nesting in urban parks; (b) pigeons nesting in other urban infrastructures like buildings.

From each captured bat and pigeon, a pool of trachea, intestines, lungs, and kidney was collected as a combined lysate for analysis. Tissue samples were obtained through sterile dissection, ensuring minimal risk of contamination. For bats, additional samples of spleen and fecal material were also collected, with fecal samples obtained directly from the transportation containers used during specimen transfer. All samples were processed immediately following collection or stored at −80 °C for further analysis.

A total number of 17 human sputum samples were provided from “Hospital de Infectología de Guayaquil” located in Guayaquil, collected during 2023 and 2024. All the individuals were positive for histoplasmosis by polymerase chain reaction (PCR) and residents of Guayaquil city.

### Microbiological cultures

To isolate *H. capsulatum*, tissue samples underwent mechanical maceration under sterile conditions and were inoculated onto three types of culture media: selective agar, brain heart infusion, and Sabouraud agar. A dual-temperature incubation strategy was employed to enhance the detection. Primary cultures were incubated at 28–30 °C, while additional Sabouraud agar plates were incubated at 37 °C to promote the pleomorphic transformation characteristic of the fungus. Cultures were monitored systematically, with initial evaluations conducted after 10 days post-inoculation. Observations continued for 30 days to account for the slow growth of fungal colonies. Colonies with morphological features consistent with *H. capsulatum* were sub cultured onto Sabouraud agar for further characterization. Microscopic examination was performed to identify key fungal structures.

### DNA extraction and nested-PCR

Pooled tissues samples from bats and pigeons, and sputum samples from humans, were processed for genomic DNA extraction. DNA was extracted using the Invitrogen™ PureLink genomic DNA Kit, following the manufacturer’s protocol. For bat and pigeon samples, the pooled tissues were previously homogenized.

The *hcp100* gene was chosen as a marker for *H. capsulatum* identification because it offers high sensitivity and specificity (26, 27). Moreover, The *Hcp100* gene encodes a 100 kDa coactivator protein that is unique to *H. capsulatum*, making it an ideal molecular marker. Its sequence is highly conserved within the species, reducing the risk of cross-reactivity with other fungi or host DNA (27). While ITS1-based qPCR may sometimes be more sensitive for fungi cultures, Hcp100 based nested PCR remains a robust choice for diagnosis directly from animal/human samples and is widely validated in the literature and used in multiple bat studies (26, 28, 29).

Nested PCR was carried out as follows, using *hcp100* gene as target (see Supplementary Table 1 for details about primers used in the study). First PCR was prepared in a 50 μL volume containing 1X Pfu DNA polymerase buffer with MgSO4, 0.2 mM dNTPs, 0.8 μM of primers (HcI/HcII), and 1.25 U Pfu DNA polymerase. Thermal cycling parameters included an initial denaturation at 94 °C for 5 min, followed by 35 cycles of denaturation at 94 °C for 30 s, annealing at 61 °C for 30 s, and extension at 72 °C for 60 s, concluding with a final extension at 72 °C for 5 min. The secondary PCR was conducted using internal primers (HcIII/HcIV) with 4 μL of the primary PCR product as the template. Reagent concentrations remained consistent with the primary reaction. The cycling conditions were modified to 30 cycles with an elevated annealing temperature of 65 °C. Amplification products were visualized on 2% agarose gels, with expected product sizes of 391 bp and 210 bp for the primary and secondary reactions, respectively.

### DNA Sanger sequencing of hcp100 gene amplicons

Positive PCR products from the secondary reaction were purified using Exonuclease I and FastAP Thermo sensitive alkaline phosphatase (Thermo Fisher Scientific, United States). Sequencing reactions were performed with the BigDye Terminator Cycle Sequencing Kit v 3.1 (Applied Biosystems, United States), and then purified by gel filtration using Sephadex G-100 (Cytiva, United States). Finally, samples were analyzed by capillary electrophoresis in an ABI3500 Genetic Analyzer (Applied Biosystems, United States). Amplicons were processed by the Sanger Sequencing Service at the Universidad de las Américas, Ecuador. Electropherograms were visualized, and quality was assessed with the Geneious Prime v2025.0 bioinformatics package.^1^ Forward and reverse reads were assembled into contigs.

### Phylogenetic analysis

*Hcp100* gene sequences from 17 human, 46 bat, and 8 pigeon samples were compared with 58 reference sequences representing known *Histoplasma* phylogenetic species (Supplementary Table 2). Contigs were identified using Basic Local Alignment Tool Nucleotide (BLAST-N) searches against the NCBI nr/nt database. Sequences were aligned with ClustalW (30). Phylogenetic analysis was conducted in IQ-TREE, with the best-fit substitution model (K2P + G4) selected using ModelFinder according to the Bayesian Information Criterion (BIC) (31). A maximum-likelihood tree was then inferred with 1,000 bootstrap replicates to assess node support. Tree visualization was performed using TreeViewer 2.2.0 (26).

### Statistical analysis

An analysis of relative and absolute frequency was performed to characterize the distribution of *H. capsulatum* in samples. For the risk factor analysis, data from bats and pigeons were analyzed separately. Either for bats or pigeons, odds ratios (OR) with 95% Confidence Intervals (CI) (Wilson score method, adjusted for species clustering in bats assuming an intraclass correlation coefficient of 0.005) and *p*-values were calculated using both chi-square and Fisher’s exact tests for all variables. All analyses were conducted using Epi Info version 7.2.6.0.

## Results

### Prevalence of *Histoplasma capsulatum* in bats and pigeons and risk factor analysis

The prevalence of *H. capsulatum* in bats and pigeons was assessed using nested PCR. As shown in Table 1, a prevalence of 13.11% (8/61; 95% CI: 10.54–15.68) in pigeons and 21.14% (45/213; 95% CI: 15.43–26.85) in bats was found. For bats, there was variability of prevalence across species, as it is detailed in Table 1, with values ranging from zero to more than 65%. Additionally, seven positives *H. capsulatum* cultures were obtained from bats samples, further confirmed by PCR.

The risk factor analysis for *H. capsulatum* carriage in bats is detailed in Table 2A, based in the variables and categories described in the methods. Among the variables analyzed, the capture zone was found to be statistically associated with the presence of the fungus in bats (*p =* 0.016). Specifically, bats captured in forests and caves showed higher positivity rates compared to other zones. However, no significant associations were observed for sex or origin (urban vs. rural settings). For pigeons, the risk factor analysis of *H. capsulatum* carriage is detailed in Table 2B, based in the variables and categories described in the methods. Among the variables analyzed, no significant association was observed neither for sex nor for capture zone (nesting in parks or in other infrastructures). However, as bats and pigeons sampling was done at convenience we cannot rule out sampling bias in our study. In this sense, the results of this risk factor analysis must be taken with caution and just as a preliminary approach.

**Table 2:** **(A)** Risk factors analysis for *Histoplasma capsulatum* infection in the 213 bats included in the study (* statistically significant association; *p*-values correspond both chi-square and Fisher test). **(B)** Risk factors analysis for *Histoplasma capsulatum* infection in the 61 pigeons included in the study (*p*-values correspond to the Fisher test) (ND: not determined).

Risk factors		*H. capsulatum* (+)	*H. capsulatum* (−)	OR (95% CI)	*p*-value (Chi-square test)	*p*-value (Fisher test)
**(A)**						
Sex	Male	22	80	0.95 (0.49–1.84)	0.88	1.00
	Female	23	88			
Origin	Urban	20	60	1.44 (0.74–2.81)	0.28	0.30
	Rural	25	108			
Capture zone	Forest	14	28	ND	0.016*	ND
	Park	10	35			
	Houses	8	41			
	Cave	6	8			
	River	6	14			
	Livestock pen	1	13			
	Trees	0	4			
	Warehouse	0	13			
	Orchard	0	12			
**(B)**						
Sex	Male	4	26	0.96 (0.22–4.26)	1.00	1.00
	Female	4	27			
Capture Zone	Nesting in infrastructure	6	51	0.12 (0.01–0.99)	0.13	0.08
	Nesting in parks	2	2	0.12 (0.01–0.99)		

### Phylogenetic analysis of *Histoplasma capsulatum* sequences from humans, pigeons, and bats origin

**Figure 2:**
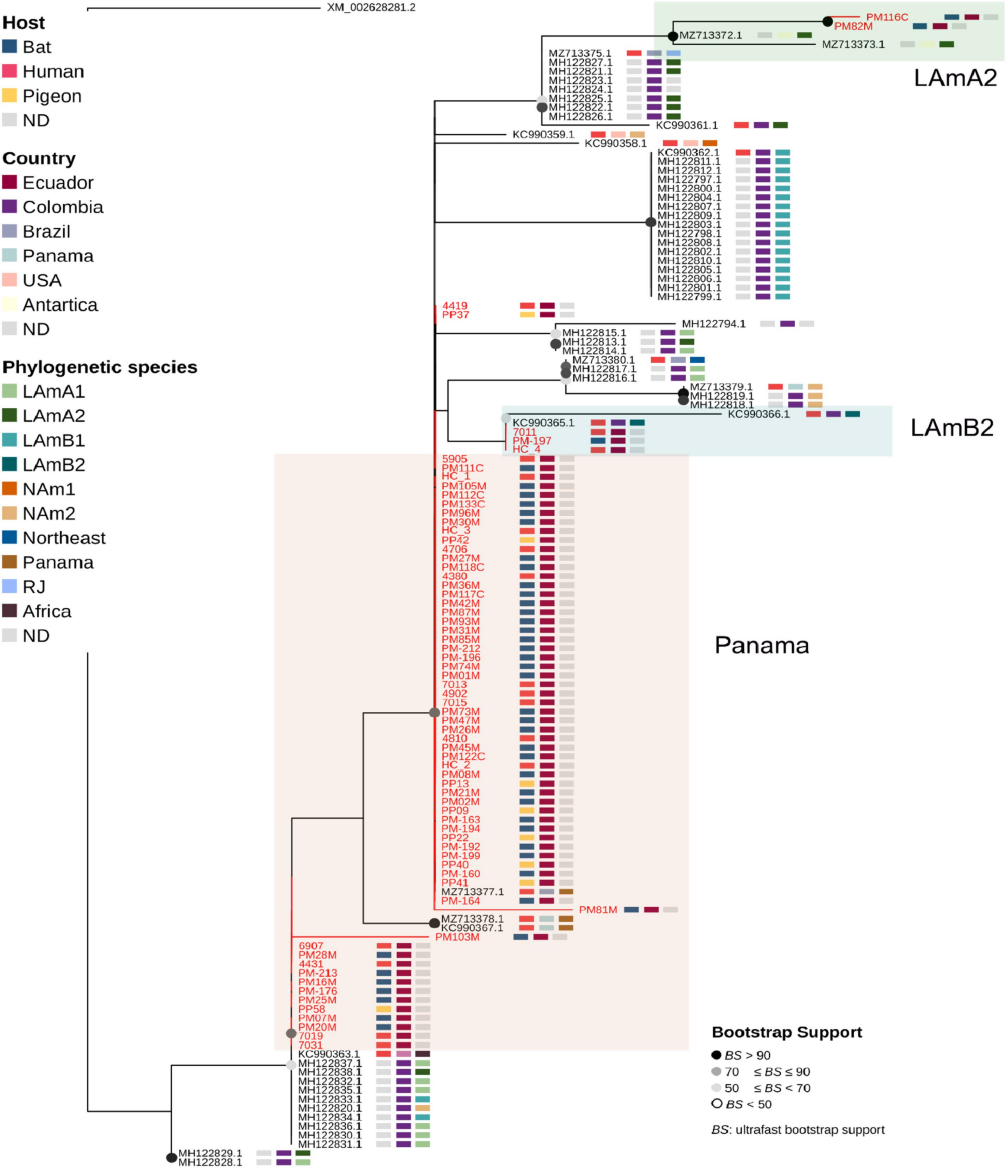
Maximum likelihood phylogeny constructed using the amplicon sequenced of the *hcp100* gene, including sequences obtained from human, bat (PM), and pigeon (PP) samples collected in this study (all highlighted in red) and those available in public databases (black text); the tree was rooted with a sequence of *Blastomyces gilchristii* as an outgroup. Shown for each sequence in rectangles are: Host type, geographic location (country) and Phylogenetic species.

The maximum-likelihood phylogenetic analysis of *H. capsulatum* sequences (Figure 2) revealed that sequences from this study clustered into three distinct phylogenetic clades. The first one, LamA2 (highlighted in green), included two bat-derived sequences from our study, forming a well-supported clade. The second one, LamB2, exhibited lower bootstrap support and comprised one bat sequence along with two human sequences from our study, as well as two isolates from humans reported in Colombia. The third and most prominent cluster corresponded to the Panama lineage, which contained the majority of sequences from this study, including those from bats, pigeons, and humans, alongside with previously reported isolates from Brazil and Panama. Accession numbers for all the sequences are provided in Supplementary Table 2.

## Discussion

This study shows a high prevalence of 21.14% for *H. capsulatum* infection affecting 10 different species of bats (*Molossus molossus, Carollia perspicillata, Glossophaga soricina, Carollia castanea, Sturnira bakeri, Artibeus lituratus, Anoura peruana, Carollia brevicauda, Desmodus rotundus,* and *Artibeus fraterculus*) from several urban and rural locations within the province of Guayas in the Coastal Region of Ecuador. Also, a high prevalence of 13.11% was found in pigeons that were exclusively collected from the city of Guayaquil in Guayas Province (the most populated city of Ecuador). Moreover, the isolation of *H. capsulatum* cultures was possible from bats samples, suggesting the spread of the pathogen from these animal reservoirs, although further research is needed to confirm this fact also for pigeons. Nevertheless, our findings suggest that Guayas Province (including the city of Guayaquil) is an endemic area for *H. capsulatum* within Latin America, with high prevalence values in animal reservoirs as previously reported in bats and pigeons from Mexico, Brazil, and Peru (32–35).

Although species-specific disparities were found in bats, further research would be needed to address a potential pattern for *H. capsulatum* prevalence across species, especially considering potential bias in our study due to the convenience sampling strategy used. In this sense, a larger and statistically representative sampling for every specie would be needed to study potential bat species patterns. The risk factor analysis confirmed a higher prevalence of *H. capsulatum* in caves and forest. Although these results align with trends observed across Latin America (where histoplasmosis outbreaks have been associated to anthropogenic perturbations of caves and forest areas), this analysis should be taken with caution due potential bias in our convenience sampling strategy (32, 36). Nevertheless, the finding of *H. capsulatum* in bats in forest areas around Guayaquil city in Guayas Province is worrisome and underscore potential recent routes of transmission to human link to the recent deforestation activity for urbanization in this city. This city has experience an explosive urbanization in the last decade. Moreover, deforestation has also taken place in rural areas to develop agriculture/aquaculture. Those events would have caused exposure of humans and domestic animals to wildlife, including bats, facilitating the spread of zoonotic diseases like histoplasmosis (37, 38).

The phylogenetic analysis (including *H. capsulatum* sequences from bat, pigeon and human origin from Guayas Province) shows a close genetic proximity with several Latin American lineages, with Panama lineage representing the larger branch, highlighting the genetic diversity in the region (13, 20). These results support previous studies linking South American strains to regional biogeographic patterns (9, 10, 14, 39). The Panama clade included most of the sequences from bats, pigeons and humans from our study, suggesting cross-species transmission of *H. capsulatum* (20). This fact is also supported by the lack of species specific branching patterns in the phylogenetic tree. So far, our phylogenetic analysis suggests the zoonotic spillover of *H. capsulatum* from bats and pigeons to humans in Guayas Province.

Guayaquil city within Guayas Province is the most populated city in Ecuador with more that 3 million inhabitants and accounts for the highest number of HIV cases in the country (21, 40, 41). Histoplasmosis is a critical driver of morbidity and mortality in immunocompromised populations like HIV patients, particularly due to delayed diagnosis and nonspecific symptomatology overlapping with tuberculosis (42, 43). The presence of *H. capsulatum* in bats not only in rural areas of Guayas, but also in Guayaquil city, and also infected pigeons, underscores a potential threat for fungal transmission from this synanthropic fauna. In this context, and integrative One Health management acknowledging the zoonotic transmission of histoplasmosis is recommended; this strategy should include a trans-disciplinary educational approach involving environmental scientist, wildlife biologist and community leaders and local authorities from either rural or urban areas to make general population aware of the risk and for instance to improve very basic biosecurity measures for safe removal of pigeons and bat. Moreover, from the clinical diagnosis perspective, our results also support the need to implement surveillance programs based of molecular detection by PCR rather than fungal culture for a sensitive, rapid and point of care diagnosis as it has been already reported for other pathogens (44).

Our study has several limitations that we would like to acknowledge. First, our sample collection was done at convenience and sample bias cannot be excluded; in this sense, the prevalence values reported and the results of the risk factor analysis should be taken with caution and only as preliminary findings; further research with larger sample size is needed to establish the true prevalence of *H. capsulatum* in pigeons and across different species of bats. Moreover, the risk factor analysis was done taking bats as a whole group due to the small sample size, so we cannot rule out that species specific patterns could be found with larger sample sizes for the variables analyzed. Second, the *hcp100* gene used in this study provided a preliminary view of the phylogenetic structure of *H. capsulatum* across humans and animal reservoirs in Guayas Province. While the reasons to choose this target are detailed in the methods, future studies employing multi locus sequencing or whole-genome analyses are necessary to delineate finer evolutionary relationships for *H. capsulatum* isolates across species. Third, we could not access to the epidemiological information of the human patients included in our study, or running additional surveys, due to the limitations in the IRB approval, so a risk factor analysis for occupational exposure to animal reservoirs was not possible.

In conclusion, the high prevalence of *H. capsulatum* in pigeons and bats from Guayas Province, endemic for human histoplasmosis, point outs the urgent need of a surveillance program based on a One Health approach. Moreover, educational programs for risk population like HIV patients should be considered for a successful histoplasmosis control and prevention program.

## Data Availability

The datasets presented in this study can be found in online repositories. The names of the repository/repositories and accession number(s) can be found in the article/Supplementary material.

## Footnotes

^1^ https://www.geneious.com/
