# Supplementary material for: High prevalence of Histoplasma capsulatum in bats and pigeons is linked to human histoplasmosis in an endemic area of Ecuador
Source: Front Vet Sci. 2025 Sep 9;12:1613841. doi: 10.3389/fvets.2025.1613841 (PMC12454044; doi:10.3389/fvets.2025.1613841)
Supplement: Supplementary file 1 [file Table_1.docx]

**Supplementary Table 1. Primers sequences and amplicon sizes for nested PCR used for identification of *Histoplasma capsulatum***

| **Gene** | **primer name** | **Sequence 5’3’** | **size** |
| --- | --- | --- | --- |
| **Hcp100** | HcI | GCGTTCCGAGCCTTCCACCTCAAC | 391pb |
|  | HcII | ATGTCCCATCGGGCGCCGTGTAGT |  |
|  | HcIII | GAGATCTAGTCGCGGCCAGGTTCA | 210bp |
|  | HcIV | AGGAGAGAACTGTATCGGTGGCTTG |  |

**References**

Bialek R, Feucht A, Aepinus C, et al. Evaluation of two nested PCR assays for detection of Histoplasma capsulatum DNA in human tissue. Journal of Clinical Microbiology. 2002 May;40(5):16441647. DOI: 10.1128/jcm.40.5.16441647.2002.

da Silva JA, Scofield A, Barros FN, de Farias DM, RietCorrea G, Bezerra Júnior PS, Santos TFS, Tavares GSF, Trevelin LC, da Paz GS, Cerqueira VD. Molecular detection of Histoplasma capsulatum in bats of the Amazon biome in Pará state, Brazil. Transbound Emerg Dis. 2021 Mar;68(2):758766. doi: 10.1111/tbed.13740. Epub 2020 Aug 4. PMID: 32686315.
