# Supplementary material for: High prevalence of Histoplasma capsulatum in bats and pigeons is linked to human histoplasmosis in an endemic area of Ecuador
Source: Front Vet Sci. 2025 Sep 9;12:1613841. doi: 10.3389/fvets.2025.1613841 (PMC12454044; doi:10.3389/fvets.2025.1613841)
Supplement: Supplementary file 2 [file Table_2.docx]

**Supplementary Table 2. List of the *Histoplasma capsulatum* reference sequences used in this work**

| **Isolate** | **Accession Number** | **Organism** | **Host** | **Country** | **Reference** |
| --- | --- | --- | --- | --- | --- |
| COL_H_068 | MH122838.1 | *Histoplasma capsulatum* | Homo sapiens | Colombia | Gómez, et al., 2019 |
| COL_S_3 | MH122837.1 | *Histoplasma capsulatum* |  | Colombia | Gómez, et al., 2019 |
| COL_S_2 | MH122836.1 | *Histoplasma capsulatum* |  | Colombia | Gómez, et al., 2019 |
| COL_S_1 | MH122835.1 | *Histoplasma capsulatum* |  | Colombia | Gómez, et al., 2019 |
| COL_H_064 | MH122834.1 | *Histoplasma capsulatum* | Homo sapiens | Colombia | Gómez, et al., 2019 |
| COL_H_047 | MH122833.1 | *Histoplasma capsulatum* | Homo sapiens | Colombia | Gómez, et al., 2019 |
| COL_H_044 | MH122832.1 | *Histoplasma capsulatum* | Homo sapiens | Colombia | Gómez, et al., 2019 |
| COL_H_021 | MH122831.1 | *Histoplasma capsulatum* | Homo sapiens | Colombia | Gómez, et al., 2019 |
| COL_H_019 | MH122830.1 | *Histoplasma capsulatum* | Homo sapiens | Colombia | Gómez, et al., 2019 |
| COL_H_053 | MH122829.1 | *Histoplasma capsulatum* | Homo sapiens | Colombia | Gómez, et al., 2019 |
| COL_H_039 | MH122828.1 | *Histoplasma capsulatum* | Homo sapiens | Colombia | Gómez, et al., 2019 |
| COL_H_062 | MH122827.1 | *Histoplasma capsulatum* | Homo sapiens | Colombia | Gómez, et al., 2019 |
| COL_H_055 | MH122826.1 | *Histoplasma capsulatum* | Homo sapiens | Colombia | Gómez, et al., 2019 |
| COL_H_041 | MH122825.1 | *Histoplasma capsulatum* | Homo sapiens | Colombia | Gómez, et al., 2019 |
| COL_H_034 | MH122824.1 | *Histoplasma capsulatum* | Homo sapiens | Colombia | Gómez, et al., 2019 |
| COL_H_033 | MH122823.1 | *Histoplasma capsulatum* | Homo sapiens | Colombia | Gómez, et al., 2019 |
| COL_H_025 | MH122822.1 | *Histoplasma capsulatum* | Homo sapiens | Colombia | Gómez, et al., 2019 |
| COL_H_013 | MH122821.1 | *Histoplasma capsulatum* | Homo sapiens | Colombia | Gómez, et al., 2019 |
| COL_H_038 | MH122820.1 | *Histoplasma capsulatum* | Homo sapiens | Colombia | Gómez, et al., 2019 |
| COL_H_005 | MH122819.1 | *Histoplasma capsulatum* | Homo sapiens | Colombia | Gómez, et al., 2019 |
| COL_H_001 | MH122818.1 | *Histoplasma capsulatum* | Homo sapiens | Colombia | Gómez, et al., 2019 |
| COL_H_048 | MH122817.1 | *Histoplasma capsulatum* | Homo sapiens | Colombia | Gómez, et al., 2019 |
| COL_H_004 | MH122816.1 | *Histoplasma capsulatum* | Homo sapiens | Colombia | Gómez, et al., 2019 |
| COL_H_024 | MH122815.1 | *Histoplasma capsulatum* | Homo sapiens | Colombia | Gómez, et al., 2019 |
| COL_H_015 | MH122814.1 | *Histoplasma capsulatum* | Homo sapiens | Colombia | Gómez, et al., 2019 |
| COL_H_014 | MH122813.1 | *Histoplasma capsulatum* | Homo sapiens | Colombia | Gómez, et al., 2019 |
| COL_H_042 | MH122812.1 | *Histoplasma capsulatum* | Homo sapiens | Colombia | Gómez, et al., 2019 |
| COL_H_056 | MH122811.1 | *Histoplasma capsulatum* | Homo sapiens | Colombia | Gómez, et al., 2019 |
| COL_H_040 | MH122810.1 | *Histoplasma capsulatum* | Homo sapiens | Colombia | Gómez, et al., 2019 |
| COL_H_035 | MH122809.1 | *Histoplasma capsulatum* | Homo sapiens | Colombia | Gómez, et al., 2019 |
| COL_H_032 | MH122808.1 | *Histoplasma capsulatum* | Homo sapiens | Colombia | Gómez, et al., 2019 |
| COL_H_029 | MH122807.1 | *Histoplasma capsulatum* | Homo sapiens | Colombia | Gómez, et al., 2019 |
| COL_H_018 | MH122806.1 | *Histoplasma capsulatum* | Homo sapiens | Colombia | Gómez, et al., 2019 |
| COL_H_017 | MH122805.1 | *Histoplasma capsulatum* | Homo sapiens | Colombia | Gómez, et al., 2019 |
| COL_H_016 | MH122804.1 | *Histoplasma capsulatum* | Homo sapiens | Colombia | Gómez, et al., 2019 |
| COL_H_012 | MH122803.1 | *Histoplasma capsulatum* | Homo sapiens | Colombia | Gómez, et al., 2019 |
| COL_H_006 | MH122802.1 | *Histoplasma capsulatum* | Homo sapiens | Colombia | Gómez, et al., 2019 |
| COL_H_008 | MH122801.1 | *Histoplasma capsulatum* | Homo sapiens | Colombia | Gómez, et al., 2019 |
| COL_H_057 | MH122800.1 | *Histoplasma capsulatum* | Homo sapiens | Colombia | Gómez, et al., 2019 |
| COL_H_007 | MH122799.1 | *Histoplasma capsulatum* | Homo sapiens | Colombia | Gómez, et al., 2019 |
| COL_H_036 | MH122798.1 | *Histoplasma capsulatum* | Homo sapiens | Colombia | Gómez, et al., 2019 |
| COL_H_066 | MH122797.1 | *Histoplasma capsulatum* | Homo sapiens | Colombia | Gómez, et al., 2019 |
| COL_H_020 | MH122794.1 | *Histoplasma capsulatum* | Homo sapiens | Colombia | Gómez, et al., 2019 |
| H59 | KC990362.1 | *Histoplasma capsulatum* | Homo sapiens | Colombia | Kasuga et al., 2003 |
| 24.11 | MZ713375.1 | *Histoplasma capsulatum* | Homo sapiens | Brazil | Moreira et al., 2022 |
| 01.16 | MZ713380.1 | *Histoplasma capsulatum* | Homo sapiens | Brazil | Moreira et al., 2022 |
| G217B | MZ713379.1 | *Histoplasma capsulatum* | Homo sapiens | Panama | Moreira et al., 2022 |
| G184A | MZ713378.1 | *Histoplasma capsulatum* | Homo sapiens | Panama | Moreira et al., 2022 |
| 39942 | MZ713377.1 | *Histoplasma capsulatum* | Homo sapiens | Brazil | Moreira et al., 2022 |
| S268B | MZ713373.1 | *uncultured Histoplasma capsulatum* | Homo sapiens | Antarctica: Drake Passage | Moreira et al., 2022 |
| F47 | MZ713372.1 | *uncultured Histoplasma capsulatum* | Homo sapiens | Antarctica: Drake Passage | Moreira et al., 2022 |
| H69 | KC990366.1 | *Histoplasma capsulatum* | Homo sapiens | Colombia | Kasuga et al., 2003 |
| H66 | KC990365.1 | *Histoplasma capsulatum* | Homo sapiens | Colombia | Kasuga et al., 2003 |
| H67 | KC990361.1 | *Histoplasma capsulatum* | Homo sapiens | Colombia | Kasuga et al., 2003 |
| H18 | KC990359.1 | *Histoplasma capsulatum* | Homo sapiens | USA | Kasuga et al., 2003 |
| RMSCC1001 | KC990358.1 | *Histoplasma capsulatum* | Homo sapiens | USA | Kasuga et al., 2003 |
| H81 | KC990367.1 | *Histoplasma capsulatum* | Homo sapiens | Panama | Kasuga et al., 2003 |
| H91 | KC990363.1 | *Histoplasma capsulatum* | Homo sapiens | Liberia | Kasuga et al., 2003 |
| HC_4 |  | *Histoplasma capsulatum* | Homo_sapiens | Ecuador | This work |
| HC_1 |  | *Histoplasma capsulatum* | Homo_sapiens | Ecuador | This work |
| HC_2 |  | *Histoplasma capsulatum* | Homo_sapiens | Ecuador | This work |
| HC_3 |  | *Histoplasma capsulatum* | Homo_sapiens | Ecuador | This work |
| 4380 |  | *Histoplasma capsulatum* | Homo_sapiens | Ecuador | This work |
| 4419 |  | *Histoplasma capsulatum* | Homo_sapiens | Ecuador | This work |
| 4706 |  | *Histoplasma capsulatum* | Homo_sapiens | Ecuador | This work |
| 4431 |  | *Histoplasma capsulatum* | Homo_sapiens | Ecuador | This work |
| 4902 |  | *Histoplasma capsulatum* | Homo_sapiens | Ecuador | This work |
| 4810 |  | *Histoplasma capsulatum* | Homo_sapiens | Ecuador | This work |
| 7013 |  | *Histoplasma capsulatum* | Homo_sapiens | Ecuador | This work |
| 5905 |  | *Histoplasma capsulatum* | Homo_sapiens | Ecuador | This work |
| 6907 |  | *Histoplasma capsulatum* | Homo_sapiens | Ecuador | This work |
| 7011 |  | *Histoplasma capsulatum* | Homo_sapiens | Ecuador | This work |
| 7015 |  | *Histoplasma capsulatum* | Homo_sapiens | Ecuador | This work |
| 7019 |  | *Histoplasma capsulatum* | Homo_sapiens | Ecuador | This work |
| 7031 |  | *Histoplasma capsulatum* | Homo_sapiens | Ecuador | This work |
| PM01M |  | *Histoplasma capsulatum* | Bat | Ecuador | This work |
| PM02M |  | *Histoplasma capsulatum* | Bat | Ecuador | This work |
| PM07M |  | *Histoplasma capsulatum* | Bat | Ecuador | This work |
| PM08M |  | *Histoplasma capsulatum* | Bat | Ecuador | This work |
| PM16M |  | *Histoplasma capsulatum* | Bat | Ecuador | This work |
| PM20M |  | *Histoplasma capsulatum* | Bat | Ecuador | This work |
| PM21M |  | *Histoplasma capsulatum* | Bat | Ecuador | This work |
| PM25M |  | *Histoplasma capsulatum* | Bat | Ecuador | This work |
| PM26M |  | *Histoplasma capsulatum* | Bat | Ecuador | This work |
| PM27M |  | *Histoplasma capsulatum* | Bat | Ecuador | This work |
| PM28M |  | *Histoplasma capsulatum* | Bat | Ecuador | This work |
| PM30M |  | *Histoplasma capsulatum* | Bat | Ecuador | This work |
| PM31M |  | *Histoplasma capsulatum* | Bat | Ecuador | This work |
| PM36M |  | *Histoplasma capsulatum* | Bat | Ecuador | This work |
| PM42M |  | *Histoplasma capsulatum* | Bat | Ecuador | This work |
| PM45M |  | *Histoplasma capsulatum* | Bat | Ecuador | This work |
| PM47M |  | *Histoplasma capsulatum* | Bat | Ecuador | This work |
| PM73M |  | *Histoplasma capsulatum* | Bat | Ecuador | This work |
| PM74M |  | *Histoplasma capsulatum* | Bat | Ecuador | This work |
| PM81M |  | *Histoplasma capsulatum* | Bat | Ecuador | This work |
| PM82M |  | *Histoplasma capsulatum* | Bat | Ecuador | This work |
| PM85M |  | *Histoplasma capsulatum* | Bat | Ecuador | This work |
| PM87M |  | *Histoplasma capsulatum* | Bat | Ecuador | This work |
| PM93M |  | *Histoplasma capsulatum* | Bat | Ecuador | This work |
| PM96M |  | *Histoplasma capsulatum* | Bat | Ecuador | This work |
| PM103M |  | *Histoplasma capsulatum* | Bat | Ecuador | This work |
| PM105M |  | *Histoplasma capsulatum* | Bat | Ecuador | This work |
| PM111C |  | *Histoplasma capsulatum* | Bat | Ecuador | This work |
| PM112C |  | *Histoplasma capsulatum* | Bat | Ecuador | This work |
| PM116C |  | *Histoplasma capsulatum* | Bat | Ecuador | This work |
| PM117C |  | *Histoplasma capsulatum* | Bat | Ecuador | This work |
| PM118C |  | *Histoplasma capsulatum* | Bat | Ecuador | This work |
| PM122C |  | *Histoplasma capsulatum* | Bat | Ecuador | This work |
| PM133C |  | *Histoplasma capsulatum* | Bat | Ecuador | This work |
| PM-160 |  | *Histoplasma capsulatum* | Bat | Ecuador | This work |
| PM-163 |  | *Histoplasma capsulatum* | Bat | Ecuador | This work |
| PM-164 |  | *Histoplasma capsulatum* | Bat | Ecuador | This work |
| PM-176 |  | *Histoplasma capsulatum* | Bat | Ecuador | This work |
| PM-192 |  | *Histoplasma capsulatum* | Bat | Ecuador | This work |
| PM-194 |  | *Histoplasma capsulatum* | Bat | Ecuador | This work |
| PM-196 |  | *Histoplasma capsulatum* | Bat | Ecuador | This work |
| PM-197 |  | *Histoplasma capsulatum* | Bat | Ecuador | This work |
| PM-199 |  | *Histoplasma capsulatum* | Bat | Ecuador | This work |
| PM-212 |  | *Histoplasma capsulatum* | Bat | Ecuador | This work |
| PM-213 |  | *Histoplasma capsulatum* | Bat | Ecuador | This work |
| PP09 |  | *Histoplasma capsulatum* | Pigeon | Ecuador | This work |
| PP13 |  | *Histoplasma capsulatum* | Pigeon | Ecuador | This work |
| PP22 |  | *Histoplasma capsulatum* | Pigeon | Ecuador | This work |
| PP37 |  | *Histoplasma capsulatum* | Pigeon | Ecuador | This work |
| PP40 |  | *Histoplasma capsulatum* | Pigeon | Ecuador | This work |
| PP41 |  | *Histoplasma capsulatum* | Pigeon | Ecuador | This work |
| PP42 |  | *Histoplasma capsulatum* | Pigeon | Ecuador | This work |
| PP58 |  | *Histoplasma capsulatum* | Pigeon | Ecuador | This work |
